# The complete chloroplast genome of *Callicarpa macrophylla* Vahl.

**DOI:** 10.1080/23802359.2022.2134749

**Published:** 2022-10-21

**Authors:** Yu Liu, Song Guo, Jinlong Bei, Wuwei Wu

**Affiliations:** a Guangxi Botanical Garden of Medicinal Plants, Nanning, PR China; b College of Food and Biochemical Engineering, Guangxi Science and Technology Normal University, Laibin, PR China; c Key Laboratory for Zhuang and Yao Pharmaceutical Quality Biology, Guangxi Science & Technology Normal University, Laibin, PR China; d Agro-Biological Gene Research Center, Guangdong Academy of Agricultural Sciences, Guangzhou, PR China

**Keywords:** Chloroplast genome, *Callicarpa macrophylla* Vahl., phylogenetic analysis

## Abstract

*Callicarpa macrophylla* Vahl. belongs to the family Lamiaceae. Its root is a widely used Yao Medicine (YM) to treat internal and external bleeding at the Yao minority areas in southern China. Here, we provide the complete chloroplast genome of *C. macrophylla* which was collected from Laibin city in Guangxi, China. The total length of the chloroplast genome is 154,141 bp, including a large single-copy (LSC) region, a small single-copy (SSC) region, and a pair of inverted repeats (IRs) regions which are separated by the LSC and SSC, with lengths of 84,904 bp, 17,839 bp, and 25,699 bp, respectively. One hundred and thirty-one genes were identified, including 89 protein-coding genes, 34 tRNA genes, and eight rRNA genes. The overall GC content is 38%. Phylogenetic analysis revealed that *C. macrophylla* is closely related to *C. integerrima* var. *chinensis*.

The genus *Callicarpa* is a member of the Lamiaceae family, which contains around 220 species and distributes throughout the tropical and subtropical regions of Asia and Oceanica, and the *Callicarpa macrophylla* Vahl. (Martin 1794) is one of the *Callicarpa* species distributed mainly in southern China (Editorial Committee of the Flora of China 1994). *C. macrophylla* is traditionally used in India, China, and South Asia as a folk medicine. It is also called ‘Chan-Gu-Feng’ in the Yao minority areas in southern China. The whole plant of *C. macrophylla* including the root, the stem, and the leaves are used in Yao Medicine (YM) with clinical actions to stop bleeding and pain, eliminate blood stasis, and reduce swelling (Xu et al. 2015). Modern pharmacological studies reveal that several terpenoids and diterpenoids from *C. macrophylla* showed potential cytotoxic activity on human cancer cell, stimulation of neuronal cell outgrowth and inhibition of NO production in LPS-activated RAW 264.7 macrophages (Xu et al. 2015; Wang et al. 2017; Lam et al. 2021).

Many medicinal plants are very difficult to distinguish from their counterparts, and the chloroplast genome is one of the most accurate tools in plant species identification (Liu et al. 2021). However, the genetic information of *C. macrophylla* is still lacking and its phylogenetic relationships have never been well tested. To facilitate the identification of the genuine ‘Chan-Gu-Feng’ in YM, here, we sequence, assemble, and annotate the whole chloroplast genome of *C. macrophylla*.

The fresh *C. macrophylla* leaves were collected from Jinxiu Yao Autonomous County, Guangxi Province, China (N: 24°07′51.70″, E: 110°06′3.87″) and a specimen was deposited at the Herbarium of Yao Medical Hospital of Jinxiu Yao Autonomous County (identified by Song Guo, guosong0804@163.com) under the voucher number CGF202006. Total genomic DNA was extracted from approximately 10 mg of silica-dried leaf tissue by using a DNeasy Plant Mini Kit (QIAGEN, Hilden, Germany) following the manufacturer’s instructions. Six microgram of DNA was used as a template in constructing a sequencing library. Paired‐end reads of 2 × 150 bp for the sample were generated on an Illumina NovaSeq6000 platform (Illumina, San Diego, CA). Low-quality reads and adapters were removed by the FastQC software (Andrews 2010). The chloroplast genome was assembled using the program NOVOPlasty 2.7.2 (Dierckxsens et al. 2017), with the complete chloroplast genome of *C. formosana* chloroplast genome (GenBank accession number: MW252167.1) as the reference.

A total of 14,591,526 sequences were obtained for chloroplast genome assembly after the paired-end clean reads were combined. The annotation was carried out by comparing the chloroplast genomes of related species in Geneious v 11.1.5 (Biomatters Ltd, Auckland, New Zealand), and the annotation results were confirmed and modified by CPGAVAS online tool (Zuo et al. 2017; Chen et al. 2019). The annotated genomic sequence was registered into GenBank with an accession number (MW829279).

The complete chloroplast genome of *C. macrophylla* is 154,141 bp in length, consisting of a large single-copy (LSC) region, a small single-copy (SSC) region, and a pair of inverted repeats (IRs) regions with lengths of 84,904 bp, 17,839 bp, and 25,699 bp, respectively. A total of 131 unique genes were identified from the chloroplast genome, including 89 protein-coding genes, 34 tRNA genes, and eight rRNA genes. The overall GC content is 38.00%.

In order to explore the phylogenetic position and evolutionary relationship of *C. macrophylla*, a phylogenetic tree was carried out using cp genome sequences of *C. macrophylla* and other 11 complete chloroplast genomes (Figure 1). The phylogenetic tree was generated based on whole chloroplast genome sequences (Zhou et al. 2018; Chen et al. 2019) and analyzed with MEGA6 software (Koichiro et al. 2013) using maximum-likelihood (ML) method (bootstrap values were calculated out of 1000 replicates) (Hu et al. 2018). Phylogenetic analysis indicated that *C. macrophylla* is placed within a monophyletic clade including all other species of *Callicarpa* sampled here. It is difficult to establish relationships among the *Callicarpa* species due to the short branches in the phylogenetic tree, which is likely a result of low nucleotide variability between these plastomes. The *Callicarpa* clade is sister to *Ocimum tenuiflorum*, the only Lamiaceae species included in this analysis. Additionally, on this phylogenetic tree *Avicennia marina* (belongs to the Acanthaceae), Lippia and Duranta (all belong to the Verbenaceae) are clearly more distant related to *Callicarpa* species.

**Figure 1.**
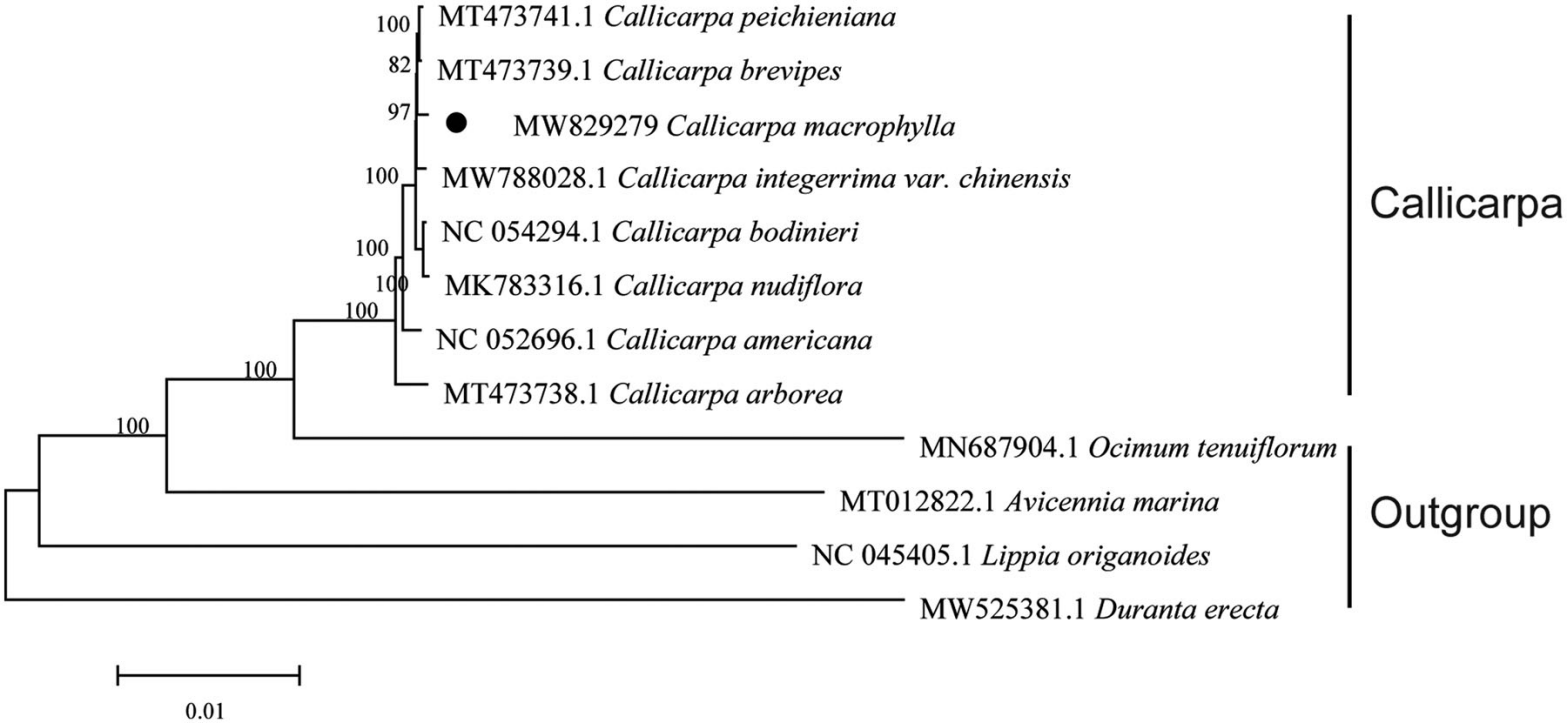
Phylogenetic placement of *C. macrophylla* resolved by maximum-likelihood method based on the complete chloroplast genome. The bootstrap values are listed on nodes.

## Data Availability

The genome sequence data can be accessed via accession number MW829279 in GenBank of NCBI at https://www.ncbi.nlm.nih.gov. The associated Bio-Project, SRA, and Bio-Sample numbers of the raw sequence data for assembling the cp genome are PRJNA715070, SRR13985461, and SAMN18325378, respectively.
